# Evaluating the Integrated Methadone and Anti-Retroviral Therapy Strategy in Tanzania Using the RE-AIM Framework

**DOI:** 10.3390/ijerph16050728

**Published:** 2019-02-28

**Authors:** Saria Hassan, Alexis Cooke, Haneefa Saleem, Dorothy Mushi, Jessie Mbwambo, Barrot H. Lambdin

**Affiliations:** 1 Yale School of Medicine, Yale University, New Haven, CT 06511, USA; 2 San Francisco Department of Psychiatry, University of California, San Francisco, CA 94118, USA; alexis.cooke@ucsf.edu; 3 Johns Hopkins Bloomberg School of Public Health, Johns Hopkins University, Baltimore, MD 21205, USA; haneefa.saleem@jhu.edu; 4 Department of Psychiatry, Muhimbili University of Health and Allied Sciences, Dar-Es-Salaam 11000, Tanzania; dorrymush@yahoo.com (D.M.); jmbwambo@gmail.com (J.M.); 5 RTI International, San Francisco, CA 94104, USA; blambdin@rti.org

**Keywords:** RE-AIM, opioid, sub-Saharan Africa, key population, HIV, integration

## Abstract

There are an estimated 50,000 people who inject drugs in Tanzania, with an HIV prevalence in this population of 42%. The Integrated Methadone and Anti-Retroviral Therapy (IMAT) strategy was developed to integrate HIV services into an opioid treatment program (OTP) in sub-Saharan Africa and increase anti-retroviral therapy (ART) initiation rates. In this paper, we evaluate the IMAT strategy using an implementation science framework to inform future care integration efforts in the region. IMAT centralized HIV services into an OTP clinic in Dar Es Salaam, Tanzania: HIV diagnosis, ART initiation, monitoring and follow up. A mixed-methods, concurrent design, was used for evaluation: quantitative programmatic data and semi-structured interviews with providers and clients addressed 4 out of 5 components of the RE-AIM framework: reach, effectiveness, adoption, implementation. Results showed high *reach:* 98% of HIV-positive clients received HIV services; *effectiveness:* 90-day ART initiation rate doubled, from 41% pre-IMAT to 87% post-IMAT (*p* < 0.001); proportion of HIV-positive eligible clients on ART increased from 71% pre-IMAT to 98% post-IMAT (*p* < 0.001). There was high *adoption* and *implementation* protocol fidelity. Qualitative results informed barriers and facilitators of RE-AIM components. In conclusion, we successfully integrated HIV care into an OTP clinic in sub-Saharan Africa with increased rates of ART initiation. The IMAT strategy represents an effective care integration model to improve HIV care delivery for OTP clients.

## 1. Introduction

The prevention, treatment and management of HIV in key populations have been recognized as a global priority if we are to reach the UNAIDS 2030 ‘End of AIDS’ target [1,2]. The evidence for using treatment as prevention for key populations is strong—the World Health Organization (WHO) recommends immediate anti-retroviral therapy (ART) initiation [3,4,5]. People who inject drugs account for 10% of HIV infections world-wide [6,7]. Yet, people who inject drugs (PWID) are less likely to receive ART than their non-drug using counterparts, with only 4% of PWID receiving ART (compared to 46% world-wide coverage) [8]. The prompt treatment of PWID is imperative to curb the spread of HIV and reduce HIV-related mortality [9,10].

At Muhimbili National Hospital, in Dar-Es-Salaam, Tanzania, an opioid treatment program (OTP) clinic provides services, including medication assisted therapy (methadone), for people who use drugs (PWUD) including injection drug users. Only 11% of HIV-positive OTP clients who are eligible for anti-retroviral therapy, initiated ART within 30 days of diagnosis, 41% within 90 days [11]. With 30,000 PWID in Dar-Es-Salaam, and an HIV prevalence of 47% among PWID compared to 7% in the general population of the city [12,13], the low ART initiation rate represented a significant performance gap that undermined the effectiveness of the OTP clinic in providing HIV treatment as prevention.

Our prior research identified factors that contribute to the low rate of ART initiation [11]. HIV care for PWUD attending the OTP clinic was provided at the HIV Care and Treatment Center by non-OTP providers, about a 20-minute walk from the OTP clinic. Reported barriers at the HIV Care and Treatment Center included the inconvenience of location, long wait times, frequent lack of reagents for CD4 testing, as well as stigmatization (for their history of drug use) by providers and other patients at the HIV Care and Treatment Center. These factors dis-incentivized OTP clients from seeking HIV care. 

The WHO and UNAIDs recommend integration of harm reduction and HIV services as a means of reaching key populations with essential HIV care [1,2]. There is strong evidence that medication assisted therapy for opioid abuse improves ART use and ART adherence among PWID [14]. There have been successful models of integrating opioid treatment with HIV service delivery described in the literature in both high-income as well as low-and-middle-income countries [15,16,17,18,19,20,21,22,23]. These models include examples of the addition of medication assisted treatment to existing HIV service delivery programs, and the addition of HIV services to existing opioid treatment programs [24,25]. 

We report here on the first initiative to integrate HIV and OTP services in sub-Saharan Africa. As there have been no prior models of care integration in this region, we engaged the community of OTP clinic providers and clients to develop an implementation strategy to integrate HIV services into the OTP clinic so as to increase ART initiation rates: The Integrated Methadone and Anti-Retroviral Therapy (IMAT) program. The objective of this paper is to comprehensively evaluate IMAT using the RE-AIM (reach, effectiveness, adoption, implementation, and maintenance) framework to inform the scale up and dissemination of similar integration efforts.

## 2. Methods

### 2.1. Setting

IMAT takes place at Muhimbili National Hospital OTP Clinic in Dar-Es-Salaam, Tanzania. This clinic opened in 2011 as the first publicly-funded opioid treatment program for PWUD in sub-Saharan Africa. The clinic has 15 providers (3 nurses, and 12 part-time psychiatrists), 1 social worker and 3 pharmacists. There is a pharmacy on site for methadone dispensing. As of December 2017, it has enrolled 1748 clients with 980 active clients. Active clients have attended the OTP clinic at least once in the past 7 days. Clients who miss more than 7 consecutive days must attend counseling at a local non-governmental organization before being referred back to the OTP clinic.

### 2.2. Eligibility Criteria for Starting ART

**Figure 1:**
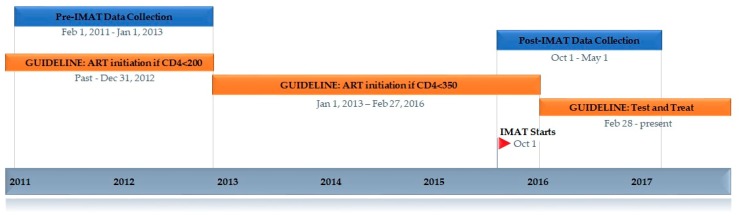
Timeline anti-retroviral therapy (ART) initiation guidelines shown in orange; data collection pre- and post-Integrated Methadone and Anti-Retroviral Therapy (IMAT) shown in blue; IMAT start date Oct 1, 2015 identified with the flag.

As shown in Figure 1, the eligibility criteria for treatment initiation for HIV positive key populations in Tanzania has evolved between 2011 and 2017. Until the end of 2012, ART initiation was based on CD4 count <200. Subsequently ART could be initiated for CD4 < 350. In February 2016, the national guidelines for key populations transitioned to test and treat—ART initiation regardless of CD4 count. 

### 2.3. Developing IMAT

We engaged the community of OTP clinic providers and clients to design a strategy to integrate HIV services into the OTP clinic and address previously identified barriers to ART initiation. We conducted in-depth interviews and surveys with OTP clinic providers and clients to address predisposing (knowledge, attitudes, beliefs, values, or perceptions that affect behavior), enabling (skills or resources that facilitate the desired change) and reinforcing (anticipated rewards and feedback following adoption of a behavior) factors to integrating HIV services into the OTP clinic (January to April 2015) [26,27,28]. For the in-depth interviews, clients were purposively sampled for gender and whether they were on anti-retroviral therapy. These results were discussed at stakeholder engagement meetings to develop the final implementation plan. Twelve clients, selected based on gender and HIV status, participated in the client engagement meeting. The qualitative data from this formative work has previously been described [29,30].

### 2.4. The IMAT Program

Standard operating procedures for HIV care at the OTP clinic were developed to reflect results of the formative evaluation and stakeholder engagement meetings. They also incorporated the Tanzanian National Guidelines for HIV care [31]. The final IMAT program consisted of multiple components that are described in Table 1. 

Predisposing factors to integration of HIV services into the OTP clinic were addressed through OTP provider education and OTP client sensitization. OTP clinic providers were trained in comprehensive HIV care and treatment through a 6-day training that covered HIV testing and counseling, assessing ART eligibility, first and second-line ART regimens, ART initiation, monitoring and adherence counseling. All trainings took place between May and October 2015. OTP client sensitization leveraged the existing trusting relationship between clients and OTP clinic nurses. Nurses had a private discussion with each HIV positive client to explain the HIV service integration plan, the importance of CD4 and HIV viral load testing, the importance of ART, and addressed any client questions or concerns. Enabling factors for service integration were addressed by the purchasing of a point-of-care CD4 machine, creation of appropriate and private clinic space, flexibility in scheduling of patients, and flexibility in ART dispensing modalities. CD4 testing was often a limiting factor that delayed ART initiation due to lack of reagents [30]. An Alere® point-of-care CD4 machine was purchased for the OTP clinic and staff were trained on its use. Providers felt it very important that there be flexibility in scheduling patients because of their existing large work burden [30]. Therefore, IMAT allowed providers to determine, at the beginning of each week, what days/times they would be available to provide HIV services. Nurses used an existing “flagging” system (used also for HIV-negative clients) to alert identified clients that they must see their provider prior to obtaining their daily methadone. To address client reluctance to receive their ART at the clinic, clients had the choice of receiving their ART at the pharmacy window, via directly observed therapy at the private nurse’s station, or via monthly supply taken at home.

To enable the reinforcing of the standard operating procedures and HIV management guidelines, a Laboratory Information Management System (LIMS) was created. This Microsoft Access-based LIMS was created to track all OTP clients, and test results; with the ability of alerting providers of patients needing follow up. IMAT implementation started in October 2015 and is ongoing.

### 2.5. Laboratory Data Collection

Baseline laboratory studies including complete blood count (white blood cell count, hemoglobin/hematocrit, platelet count), liver function tests (AST/ALT), creatinine, hepatitis B and C status were all routinely drawn on all patients prior to ART initiation. This was the case before and after IMAT implementation. Before IMAT, these tests were drawn at the HIV Care and Treatment Center. After IMAT, the blood for these analyses was drawn at the OTP Clinic. In both cases, the tests were done at the Muhimbili National Laboratory.

CD4 count was required to determine treatment eligibility until 2016. Before IMAT, blood for the CD4 count was drawn at the HIV Care and Treatment Center and sent to the Muhimbili National Laboratory for analysis. After IMAT, CD4 count was conducted using point-of-care machine

Viral load was drawn 6 months post treatment initiation in all clients before and after IMAT. Before IMAT, blood was drawn at the HIV Care and Treatment Center and analysis completed at the Muhimbili National Laboratory. After IMAT, blood was drawn at the OTP clinic and samples were sent to another local hospital—Temeke Hospital for analysis. Transportation of samples, and return of test results, was via BodaBoda (motorcycle transport). 

### 2.6. Evaluation Framework

The RE-AIM framework provides structured evaluation of the public health impact of interventions to facilitate wide-scale dissemination and implementation [32,33]. RE-AIM has been widely applied to HIV-related public health interventions world-wide [34,35,36]. RE-AIM has the benefit of employing an ecological approach to evaluation, incorporating individual-level and setting-level factors. The five key components of RE-AIM are reach, effectiveness, adoption, implementation and maintenance. In Table 2, we describe items within each of the four RE-AIM domains that we evaluated using a concurrent mixed methods approach. Maintenance was not assessed at the time of this evaluation. 

Table 2 lists the RE-AIM dimensions and items within each dimension that are evaluated for IMAT. Programmatic and qualitative data were used to assess items within each domain.

### 2.7. Quantitative Data for Evaluation

Sample and procedures: The sample included all HIV-positive OTP clients (n = 136). Programmatic data for these clients between October 2015 and May 2017, were collected from log books that were used by providers at the clinic to track interactions with all HIV-positive clients and from client HIV Care and Treatment Registration cards: provider visits, test results, ART initiation date and regimen, follow up and reasons for missed appointments. Data from the log books were entered into the Laboratory Information Management System (LIMS). For the purposes of this analysis, clients who default are those who stop attending the OTP clinic and do not re-engage in care within the study period. 

Analysis: Data for this analysis were collected pre and post IMAT implementation; IMAT started in October 2015. “Pre-IMAT” refers to data before IMAT implementation (February 2011 to January 2013) and details on ART initiation rates during this period have been previously described [11]. “Post-IMAT” data between October 2015 and May 2017 (Figure 1). For “Reach” programmatic data was reviewed to determine the number and characteristics of OTP clients who were participating in IMAT. For “Effectiveness,” the proportion of HIV-positive eligible clients on ART post-IMAT was determined for the entire sample population using programmatic data; eligibility was determined based on relevant criteria at the time of ART initiation (Figure 1). Time-to-ART initiation was determined for the group of clients who were initiated on ART between Oct 2015 and February 2016, based on the fourth edition national guidelines that stated ART should be initiated for clients with CD4 < 350 cells/µl (total n = 20) [31]. The time from the date of CD4 test to the date of ART initiation was determined. This allowed us to compare time-to-ART initiation post-IMAT, to pre-IMAT data where ART eligibility was also based on CD4 count. In February 2016, Tanzanian national guidelines changed: key populations were now to be treated with ART regardless of CD4 count [38]. The group of OTP clients who became eligible based on the new test-and-treat guidelines (n = 28) were not comparable to the pre-IMAT group due to eligibility independent of CD4 count. Pre-IMAT data were derived from routine programmatic and clinical data on clients enrolled at the OTP clinic from February 2011 to January 2013 (n = 119); during this study period, clients were eligible if their CD4 count was less than 200 cells/µl (n = 17) [11]. The chi-squared test of association was used to compare: (1) proportion of HIV-positive eligible clients on ART post- IMAT to pre-IMAT data; and (2) proportion of clients initiated on ART within 30- and 90-days of eligibility post-IMAT to the pre-IMAT data. For “Adoption,” we assessed the number and characteristics of providers who were and were not involved with IMAT. Finally, for “Implementation” percentage of perfect delivery was assessed by reviewing the Laboratory Information Management System/programmatic data to assess the proportion of individuals with guideline-consistent CD4 testing, viral load testing, and referral to the specialized HIV Care and Treatment Center.

### 2.8. Qualitative Data for Evaluation

Sample and procedures: Semi-structured interviews were conducted with 35 HIV-positive clients and 8 providers at the OTP clinic 6 months into IMAT implementation. All providers involved with IMAT implementation were interviewed. The 35 clients were purposively sampled for gender and ART treatment status. Interviews were conducted by trained research assistants in Kiswahili, audio-recorded, transcribed, and translated. Interviews with patients focused on their experiences with the IMAT intervention and adapting to the new protocol. Providers were asked about their reaction to and opinions of the IMAT intervention including its implementation, their role in patient education, intervention procedures, and ART dispensing. 

Analysis: Transcripts were analyzed using thematic content analysis. One author (AC) coded and analyzed transcripts using Dedoose (Version 7.0.23, SocioCultural Research Consultants, LLC, Los Angeles, CA, USA,2016: web application for managing, analyzing, and presenting qualitative and mixed method research data; Los Angeles, CA: Sociocultural Research Consultants, LLC). Themes were then categorized based on their relevance to each of the relevant domains of reach, effectiveness, adoption, and implementation.

This study received ethical approval from Ethical and Independent (E&I) Review Services in the United States as well as the Tanzania National Institute for Medical Research and Muhimbili University of Health and Allied Science in Tanzania. Informed consent was required for participation in cross-sectional surveys and in-depth interviews. The approvals also included the use and analysis of de-identified, programmatic data.

## 3. Results

The mixed methods results for each of the RE-AIM components as outlined in Table 2 are presented with quantitative data followed by qualitative data.

### 3.1. Reach

Our target population was all OTP clients who were HIV-positive—136 clients in October 2015 at the start of IMAT. At the end of the study period, 96% (130/136) of HIV-positive clients were engaged in IMAT (they received HIV services through the OTP clinic). Four individuals had missing information and assumed to not be engaged in IMAT, two individuals refused ART and therefore HIV care at OTP clinic. No clients were excluded from participation. The average age of the clients was 34 years, of whom 87% were male (similar to the general OTP clinic client population). No differences in average age or gender proportion or education level could be identified between participating and non-participating clients. Excluding the individuals with missing information, 4.5% (6/132) defaulted during the study period. Semi-structured interviews explained that high reach was in part attributable to clients recognizing the benefit of integrated services. One client explained:

“The current system is better than the past. In the past, we were given the medications to take at home, but many people were not taking them, and we had a calamity. Many people died. Now you have to go to take your methadone and then go to the back to take your ARVs [anti-retrovirals]. They will know that you are not taking them if you don’t show up. This system is better.”

### 3.2. Effectiveness

#### ART Initiation

We considered HIV-positive OTP clients with complete data (n = 132). Overall, the OTP client population is homogenous without significant differences in age, gender, education level, incarceration history. The proportion of HIV-positive OTP clients (eligible for treatment based on the appropriate treatment guidelines of the time) who were on ART increased from 71% (12/17) before IMAT implementation to 98% (130/132) at the end of the study period (*p* < 0.001). Six HIV-positive clients defaulted from the OTP clinic during the 20-month study period: an attrition rate of 4.5%.

**Figure 2:**
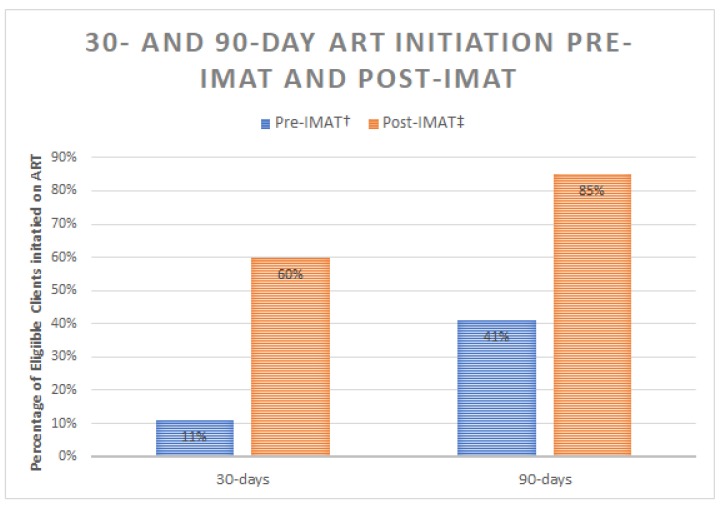
Proportion of eligible clients receiving ART pre- and post-IMAT. This figure describes the percentage of eligible opioid treatment program clients (pre-IMAT, post-IMAT) who were initiated on ART within 30 and 90 days of their eligibility. † Pre-IMAT (n = 17) Eligible based on CD4 count. ‡ Post-IMAT, (n = 20) Eligible based on CD4 count (before guideline change); 2.07-fold increase in 90-day ART initiation (*p* < 0.001).

Figure 2 shows the proportion of clients initiating ART within 30 and 90 days of eligibility. Post-IMAT, 60% (79/132) and 85% (112/132) of eligible clients were initiated on ART within 30 and 90 days respectively. Comparing pre- and post-IMAT data, there was a 2.07-fold increase in 90-day ART initiation among eligible clients (*p* < 0.0001).

### 3.3. Adoption

Providers and staff at the OTP clinic adopted the different components of the integrated model. A total of 6 providers (all three nurses and 3 out of 12 physicians), based at the Muhimbili OTP clinic were trained as HIV care and treatment clinicians. No staff was excluded from participating. Staff that participated had a long-standing interest in HIV care and treatment and had previously been involved in research initiatives at the OTP clinic. There were no other distinguishing characteristics between participating and non-participating staff. Five out of the 6 providers (3 nurses and 2 physicians) trained in HIV care and treatment administered HIV-related services under the IMAT program.

In interviews with providers, they discussed the importance of centralizing care to meet patients’ needs and improve their HIV care as key to their adoption of IMAT. As one provider stated:

“Drug users are at an increased risk of getting HIV, so it is [appropriate] for [the OTP clinic] to have HIV services integrated. It is not okay to separate HIV services from this program.”

However, the most prominent barrier to provider adoption of IMAT was the increased workload. This prevented the third physician who was trained from delivering HIV care and treatment services as part of the IMAT protocol. As one provider stated:

“This program has added the work load on us.…. We now have a lot of works to do in such a way that you cannot do an assessment on them. We have to leave other work and do this because of the shortage.”

Adoption of the Laboratory Information Management System (LIMS) database was also assessed. The purpose of the LIMS was to reinforce which HIV-related services a client needed at the time of their visit. However, adoption of the LIMS was limited to intermittent report generation. The nurses found it cumbersome and time-consuming to access the laptop and database regularly for data entry or to look up clients as they came for clinic visits. Nevertheless, it was very helpful in tracking clients, as one provider stated:

“Yes, it is very helpful because we are working as a team though we don’t get to run it directly. It reminds and guides us on who is supposed to do what…. It reminds us of who needs certain services and who is due for follow up. I am imagining it if we were doing it in the file system, it would have been such a challenge.”

### 3.4. Implementation

To assess fidelity to the IMAT protocol we used programmatic data to assess adherence to the standard operating procedures and to national HIV treatment guidelines. We assessed referral to the main CTC, CD4 testing and viral load testing per protocol. Additionally, we examined protocol adaptations made during implementation, and barriers and facilitators to IMAT implementation. 

#### 3.4.1. Referral to the CTC

In accordance with the IMAT protocol, four HIV-positive clients were referred to the main CTC, due to conditions that required specialist care. One had elevated liver function tests, the other had an elevated creatinine, one had suspected drug resistance and the fourth had co-infection with Hepatitis B and C. No clients who continued to be treated at the OTP clinic had any of the conditions requiring referral to HIV specialists.

#### 3.4.2. CD4 Testing

At the start of IMAT in 2015, CD4 defined eligibility for ART initiation, and 86 CD4 tests were conducted using the Point-of-care CD4 machine. Of those not on ART, 97% of clients received a CD4 test to determine eligibility within 5 months from the start of IMAT to guideline changes in February 2016.

#### 3.4.3. Viral Load Testing

The WHO HIV treatment guidelines recommend a viral load test every 6 to 12 months to monitor treatment and as a marker of medication adherence. During this study period, 85% of clients who were due for a viral load test received testing.

#### 3.4.4. Protocol Adaptation: Changes to Treatment Guidelines

At the start of IMAT in October 2015, Tanzanian National Guidelines for the Management of HIV and AIDS stated that even for key populations, treatment should be initiated for CD4 < 350 cells/µl [31]. In February 2016, the Tanzanian guidelines were updated to adhere to the WHO standards, now recommending all key populations should be on ART regardless of CD4 [38]. The standard operating procedures were changed to reflect this new regulation. 

#### 3.4.5. Protocol Adaptation: Client Preference

Nurses found that not all clients wanted comprehensive HIV services at the OTP clinic. Reasons for this included fear of inadvertent disclosure and stigmatization from HIV-negative clients and existing favorable relationship with an outside HIV service clinic. Therefore, while they encouraged full and comprehensive care with IMAT, the providers adapted the protocol to allow for varying levels of HIV service delivery to each client. This created three tiers of integration at the patient level: provider counseling only (minimal), with laboratory monitoring (partial), with ART dispensing (full). Of HIV-positive clients, 5% were minimally, 19% partially, and 76% fully integrated into the IMAT program. 

#### 3.4.6. Protocol Adaptation: System-Level Challenges

The goal of IMAT was to centralize all HIV-related services at the OTP clinic to minimize delays due to system-level inefficiencies. However, clinic providers still relied on outside services for baseline laboratory testing (per guidelines, hemoglobin, creatinine and liver function tests were needed, prior to initiating ART) and viral load monitoring. During IMAT implementation, these services encountered challenges. In 2016, hospital-management-led changes to the health payment structure resulted in previously exempt OTP clients needing to pay for laboratory testing at the main hospital; this cost was prohibitive. Negotiations with an alternate lab (affiliated with Muhimbili University) allowed for continuation of care for these clients. IMAT providers were able to obtain viral load testing at no charge at another government hospital Temeke Regional Hospital, about 7 kilometers away. A boda-boda (motorcycle transport) was used to deliver blood samples to Temeke Regional Hospital and return results. While this allowed for viral load testing, it introduced delays dependent on the availability of the boda-boda driver. 

#### 3.4.7. Barriers and Facilitators to Implementation

Providers felt strongly that one of the facilitators to implementation was the flexibility in the protocol to allow for scheduling HIV services based on availability of the nurse and physician. Barriers to implementation, however, included continued reliance on services outside of the OTP clinic for HIV-related care.

“The problem is with the baseline investigations results […] The clients cannot be started on ARVs if they have kidney problems […] Clients may be lost […]”

(Provider)

Clients described that barriers to implementation continued to be concerns around inadvertent HIV status disclosure and stigmatization within the OTP clinic. As one client stated,

“I am just requesting for more confidentiality […] Many people are scared of taking their [ART] medications here because they don’t want to be mocked […]”

## 4. Discussion

We have described and evaluated an implementation strategy that integrates HIV care services into a busy urban OTP clinic in Dar-Es-Salaam, Tanzania. The IMAT strategy was successful in increasing ART initiation, with 98% of HIV-positive OTP clients on ART at the end of the study period. This serves as a basis for future model development to answer the WHO call to reach key populations with integrated HIV services using a combination approach that includes harm reduction services [1,2]. Other clinics have successfully integrated HIV care and opioid treatment programs, but this is the first time, to our knowledge, that it is being reported in sub-Saharan Africa [21,39,40]. IMAT adds to the body of literature showing that integration of HIV care for PWUD is an effective approach to ensuring their timely and appropriate care and treatment [41,42,43]. 

Central to the success of IMAT is the early involvement of stakeholders to determine the predisposing, enabling and reinforcing factors for service integration specific to this setting. The IMAT protocol accommodated flexible scheduling and ART dispensing modalities that addressed provider and client concerns. This contributed to the high reach of IMAT with 98% of HIV-positive clients engaging in IMAT, as well as adoption by providers trained in HIV care (five out of six providers). This reinforces the established importance of community engagement but also highlights its feasibility among key populations in low resource settings. These are communities that have traditionally not been engaged in research but are among the most challenging for successful implementation of interventions. IMAT is an example of how the involvement of these vulnerable groups early in the implementation design process can lead to successful implementation. 

By applying a mixed methods approach to our RE-AIM evaluation, we provide a comprehensive description and evaluation of IMAT that allows for an understanding of its immediate impact and its potential for generalization. Our data and evaluation allowed for comprehensive coverage of the four RE-AIM domains that were considered. Using an established evaluation framework, allows one to understand what specific components of the intervention contributed to, or impeded, “success” [24]. This knowledge is essential for any attempt to replicate or adapt this work. Additionally, because of its ecological framing, RE-AIM allows one to understand what role context plays and therefore what adaptations might be needed when implementing the intervention in other settings. 

The RE-AIM evaluation highlighted challenges to integration of HIV services into the OTP clinic. The barriers to service integration included lack of adequate staffing, stigma and system-level barriers. These are “predisposing” and “enabling” factors that were not addressed during our formative work. While stigma was addressed initially, the RE-AIM evaluation allowed us to determine how stigma was continuing to impact implementation of the program. Fears of inadvertent disclosure posed a barrier to full integration into the IMAT program (24% of IMAT clients not fully integrate). Providers had to make adaptations to IMAT to allow clients, who feared stigmatization, to receive some (but not all) HIV related services at the OTP clinic (partial integration). Looking at “reach” alone would not have uncovered this. Studying the implementation process and the protocol adaptations that were made, and understanding qualitatively why they were made, enabled us to uncover this important and persistent barrier. As outlined in a recent systematic review, future care integration programs must address stigma at multiple levels to ensure success [44]. Our formative work had not addressed baseline laboratory and viral load testing needs (stakeholders had not predicted a change in laboratory testing fees to requiring a copay for HIV-positive OTP clients). Using the RE-AIM framework allowed us to look specifically at this aspect of implementation, independent of other domains, to understand how it impacted the program. In so doing, we can highlight the importance of future scale-up of IMAT, and its implementation in different contexts, having a sustainable and guaranteed means of covering fees not only for treatment, but for laboratory testing for initiation and monitoring of treatment. Centralizing all testing services, as free point-of-care tests, at the OTP clinic would address this “enabling” need. Alternatively, changing the payment structure for OTP clients, and improving the reliability of laboratory services at Muhimbili National Hospital, would “enable” integration as well as improve the greater health system. Lastly, while flexible scheduling was critical to provider engagement, RE-AIM allowed us a more nuanced view of adoption. This highlighted not only the need for increased staffing but the need to engage OTP providers not traditionally involved in research. Alternatively, the team could train traditional HIV providers to provide sensitive care at the OTP clinic. Importantly, implementation of IMAT in other contexts must consider not only the number of staff needed and the challenge of added workload, but the importance of having staff who are sensitized to the needs of key populations. These factors are also important to address as IMAT continues at Muhimbili and as we consider elements critical to maintenance and scale up.

Sustainability of IMAT beyond the project period is feasible but there are challenges. Trained staff are in place and a program to train additional staff in the event of staff turnover is available. IMAT has provided a model of care that allows for the continued provision of HIV related services to HIV positive clients at the OTP clinic. With current test and treat guidelines, a baseline CD4 is not required for initiation of treatment. In addition, ART continues to be provided free of charge to all HIV positive individuals. Therefore, the major foreseen barrier to sustainability is payment for baseline laboratory testing. Similar to how they support people living with HIV to receive HIV services more broadly, a local non-governmental organization provides HIV services in Dar Es Salaam pays for these laboratory investigations, allowing for the continuation of IMAT services. More permanent solutions as mentioned above should be considered to allow for dissemination and scale-up of the program. 

Despite the breadth of information garnered from using the RE-AIM framework for our evaluation, we acknowledge there are a few study limitations. Potential bias could have been introduced by the purposive sampling of participants for the engagement meetings and qualitative interviews. We were only able to engage and interview active OTP clients; therefore, we could have missed important perspectives from people who dropped out of opioid treatment or remained in opioid treatment but refused HIV testing so as not to disclose their HIV status. Additionally, the change in treatment guidelines during our project period limits the comparability of the management of HIV positive clients pre- and post-IMAT. This was overcome for the time to ART-initiation analysis by focusing only on post-intervention data that used a CD4 cut off for treatment initiation. Furthermore, we only examine ART initiation among clients deemed eligible by the appropriate criteria of the time. In addition, we acknowledge that a pre-post analysis is limited in that it is unable to account for secular trends in ART initiation, specifically in this case between 2013 and 2015. However, data on ART initiation between January 2013 and Oct 2015 was not included in the analysis due to concerns of contamination bias. Dissemination of findings from pre-IMAT data in 2013, indicating low ART-initiation rates, intensified HIV care efforts at the OTP clinic. In addition, intensive engagement of providers and participants in 2014 and 2015 through meetings, qualitative interviews, and surveys, likely influenced HIV care and treatment efforts within the OTP clinic. Therefore, we believe the pre-post analysis with our timeframes to be the best approach in this scenario. Future studies should evaluate the effectiveness of IMAT in a longitudinal study with comparison clinics. Evaluation of cost-effectiveness and maintenance of IMAT is being considered for future work. Completing these components will further facilitate the scale up and dissemination of IMAT to other OTP clinics in the region.

## 5. Conclusions

We have used established implementation science theories and frameworks to describe and evaluate a strategy to integrate HIV services into an opioid treatment program in sub-Saharan Africa. This integration resulted in increased ART initiation among PWID, addressing the need for increased access to HIV services for PWID. Recognizing that treatment as prevention among key populations is critical to curbing the spread of HIV, the IMAT strategy provides a model for HIV service integration to reach key populations that can be scaled up and adapted to other settings.

## Figures and Tables

**Table 1:** Describing Components of the IMAT Strategy.

	IMAT Strategy Components	Description
PREDISPOSING FACTORS	Opioid treatment program (OTP) provider education	OTP clinic providers trained in comprehensive HIV care and treatment They attended a 6-day training conducted by the HIV Care and Treatment Center at Muhimbili National Hospital. This training covered all aspects of managing HIV: HIV testing and counseling, assessing ART eligibility, first- and second-line ARV regimens, monitoring patients on ART, and ART initiation and adherence counseling.
	OTP client sensitization	Client sensitization to the integration of HIV care services within the OTP clinic Nurses spoke one on one with HIV-positive clients to explain process of service integration and address any questions/concerns
ENABLING FACTORS	OTP provider familiarization with IMAT Standard Operating Procedures (SOP)	The SOP described flow of care through the clinic to maintain client confidentiality Nurses provided regular HIV testing and counseling Providers initiated ART based on guidelines and ensured appropriate monitoring and follow up Reasons for referral to the main HIV Care and Treatment Center were described: pregnancy, renal disease, liver disease, or presumed drug resistance
	OTP clinic space and facilities physical adjustments	Physical adjustments to the OTP clinic space were needed to meet HIV Care and Treatment Center guidelines for managing HIV patients: locked cabinet space, extra windows for ventilation and computer access
	Alere® point-of-care CD4 machine	Purchasing of machine Providers were trained on use of the machine
	Scheduling flexibility	At the start of each week providers identified days and times they would be available to see HIV-positive clients Nurses use an existing system of “flagging” OTP clients to indicate which clients needed to be seen by a physician before obtaining their methadone. This system avoided inadvertent disclosure as it was not specific to HIV-positive clients (all OTP clients were to be seen in regular intervals by providers) Providers were available 2–4 times a week to see HIV positive patients due for follow up or to address clinical problems
	ART dispensing modalities	Different ART dispensing modalities accommodated client preference. Dispensing modalities if receiving ART at the OTP clinic: directly observed therapy (DOT) with the nurse, DOT with methadone at the pharmacy window or monthly supply from the nurse.
REINFORCING FACTORS	Laboratory information management system (LIMS)	Microsoft Access-based LIMS was created to track all OTP clients; logging testing dates and results (HIV, CD4, HIV viral load, hepatitis B, C, and tuberculosis), alerting providers on client need for HIV testing, ART initiation, CD4 or viral load testing LIMS was installed into a laptop computer in the OTP clinic Providers were trained on use of the LIMS

**Table 2:** Evaluation of the IMAT Program using RE-AIM (reach, effectiveness, adoption, implementation, and maintenance) framework.

**Reach**	
Percentage who participate: proportion of HIV-positive clients receiving HIV services through IMAT at the OTP clinic Participants excluded Participant characteristics versus nonparticipants Qualitative methods to evaluate barriers/facilitators to reach	
**Effectiveness**	
Primary outcome Measures: Proportion of newly diagnosed HIV-positive clients started on ART within 90 days Proportion of IMAT clients initiating ART Proportion of IMAT clients with CD4 on file Proportion of IMAT clients with viral load in past 6 months Differences across sub-groups of OTP clients Attrition (%): participants no longer receiving HIV services at the OTP clinic Qualitative methods: to assess barriers/facilitators to effectiveness [37]	
**Adoption**	
Staff Exclusions (% or reasons) Percentage of staff invited that participate Characteristics of staff participants vs. nonparticipating staff Use of qualitative methods to understand staff adoption	
**Implementation**	
Percentage of perfect delivery: in accordance with the IMAT SOP Adaptations made to the protocol: Protocol Adaptation: change to treatment guidelines Protocol Adaptation: patient preferences Protocol Adaptation: system-level challenges Cost of the intervention (not assessed) Qualitative methods to understand barriers/facilitators to implementation	
**Maintenance**	Not assessed; future work

## Abbreviations

ART	Anti-retroviral therapy
CTC	Care and Treatment Center
IMAT	Integrated Methadone and Anti-Retroviral Therapy
LIMS	Laboratory Information Management System
OTP	Opioid Treatment Program
PWID	People Who Inject Drugs
RE-AIM	Reach, Effectiveness, Adoption, Implementation, Maintenance
SOP	Standard Operating Procedures
